# Axillary Nodal Positivity in Early-Stage Invasive Lobular Carcinoma: Implications for Sentinel Lymph Node Biopsy Omission

**DOI:** 10.1245/s10434-026-19387-6

**Published:** 2026-03-12

**Authors:** Lorenzo Scardina, Sabatino D’Archi, Cristina Accetta, Beatrice Carnassale, Alba Di Leone, Flavia De Lauretis, Antonio Franco, Federica Gagliardi, Stefano Magno, Francesca Moschella, Maria Natale, Alejandro Martin Sanchez, Marta Silenzi, Ersilia Biondi, Enrico Di Guglielmo, Elisabetta Gambaro, Annasilvia Di Pumpo, Eleonora Petrazzuolo, Chiara Rianna, Gianluca Franceschini

**Affiliations:** Breast Unit, Department of Women, Children and Public Health Sciences, Fondazione Policlinico Universitario Agostino Gemelli IRCCS, Rome, Italy

**Keywords:** Early-stage breast cancer, Invasive lobular carcinoma, Sentinel lymph node biopsy, Axillary staging, De-escalation therapy

## Abstract

**Background:**

Recent trials suggest omission of sentinel lymph node biopsy (SLNB) for selected early-stage breast cancer patients. However, invasive lobular carcinoma (ILC) is underrepresented, and retrospective data indicate higher rates of nodal metastases, raising concerns about axillary understaging. This study aimed to evaluate the prevalence and predictors of nodal metastases in early-stage, clinically node-negative ILC.

**Methods:**

This study retrospectively analyzed 491 patients with estrogen receptor (ER)-positive/human epidermal growth factor receptor 2 (HER2)-negative, clinical T1, clinically node-negative ILC who underwent breast-conserving surgery at our institution between 2004 and 2024. The exclusion criteria ruled out neoadjuvant therapy, tumor larger than 2 cm, and metastatic disease at diagnosis or prior breast cancer.

**Results:**

Among 491 patients, 392 (79.8 %) were pN0, whereas 99 (20.2 %) had nodal metastases (pN1mi–pN3). Pathologic tumor size was significantly associated with axillary nodal involvement (*p* = 0.004). In contrast, histologic subtype was not significantly associated with nodal status (*p* = 0.15), although pleomorphic tumors demonstrated numerically higher rates of nodal involvement than classic invasive lobular carcinoma. Menopausal status was not predictive of nodal positivity (*p* = 0.96).

**Conclusions:**

Approximately one (20.2 %) in five patients with early-stage, clinically node-negative ILC harbors occult axillary nodal metastases. Pathologic tumor size emerged as the primary determinant of nodal involvement. Pleomorphic variants showed a tendency toward higher nodal burden. These findings indicate that omission of SLNB in ILC may carry a risk of axillary understaging with potential therapeutic implications. Pending evidence from prospective studies specifically designed for lobular histology, SLNB should continue to be considered an essential component of axillary evaluation in this subgroup.

Invasive lobular carcinoma (ILC) is the second most common histologic subtype of breast cancer after invasive ductal carcinoma (IDC), accounting for approximately 10 to 15 % of all breast malignancies.^1,2^ This tumor is characterized by the loss of E-cadherin, a cell-adhesion protein whose absence leads to the distinctive linear and dis-cohesive growth pattern typical of lobular histology.^3,4^ As a result, ILC often exhibits diffuse stromal infiltration and palisading cell arrangement, which differ markedly from the cohesive glandular structures observed in ductal carcinoma. This unique biologic behavior has important diagnostic and surgical implications. Due to its subtle growth and low cellular density, ILC is frequently underestimated in size and extent at both imaging and histopathologic evaluation compared with IDC.^5,6^

Even with high-resolution magnetic resonance imaging (MRI), lobular carcinomas often present as multifocal or multicentric lesions with poorly defined margins, leading to a higher rate of incomplete excision and reoperation. The same infiltrative pattern complicates clinical and imaging-based assessment of the axilla, contributing to potential under-detection of nodal disease.^7,8^ However, current imaging tools, particularly axillary ultrasound (US), continue to show limited sensitivity in identifying nodal involvement in ILC.

Another distinctive feature of ILC is its marked propensity to metastasize to axillary lymph nodes, even when the primary tumor is small or clinically indolent.^9,10^ This underscores the ongoing relevance of accurate axillary staging in the surgical management of ILC.

Importantly, ILC is a biologically heterogeneous disease encompassing classic, solid, pleomorphic, alveolar, and mixed subtypes with distinct pathologic features and clinical behavior.^11^

The recent American Society of Clinical Oncology (ASCO) guideline update on sentinel lymph node biopsy (SLNB) in early-stage breast cancer has profoundly influenced axillary surgical practice.^12^ These recommendations are largely derived from two pivotal randomized controlled trials, the SOUND and INSEMA studies, which investigated the de-escalation of axillary surgery in patients with clinically node-negative disease. The SOUND trial, published by Gentilini et al.^13^ in 2023, represented a paradigm shift by demonstrating that for carefully selected patients with early-stage breast cancer and negative axillary ultrasound findings, omission of SLNB could be considered without compromising oncologic safety. The subsequent INSEMA trial, reported by Reimer et al.^14^ in 2024, further confirmed the feasibility of this approach, reinforcing the movement toward minimally invasive and precision-guided axillary management.

The ASCO recommendations explicitly support SLNB omission only for carefully selected patients with early-stage invasive ductal carcinoma and negative axillary imaging. Therefore, these criteria cannot be directly applied to ILC.

Given the limited representation of ILC in de-escalation trials, we sought to evaluate the prevalence and determinants of nodal metastases to assess the safety of SLNB omission in this histology.

This retrospective study analyzed patients with early-stage, clinically node-negative ILC treated at our institution to determine the prevalence and predictors of nodal metastases, aiming to inform the safety and applicability of SLNB omission in this specific histologic subgroup.

## Materials and Methods

### Study Design and Setting

**Fig. 1:**
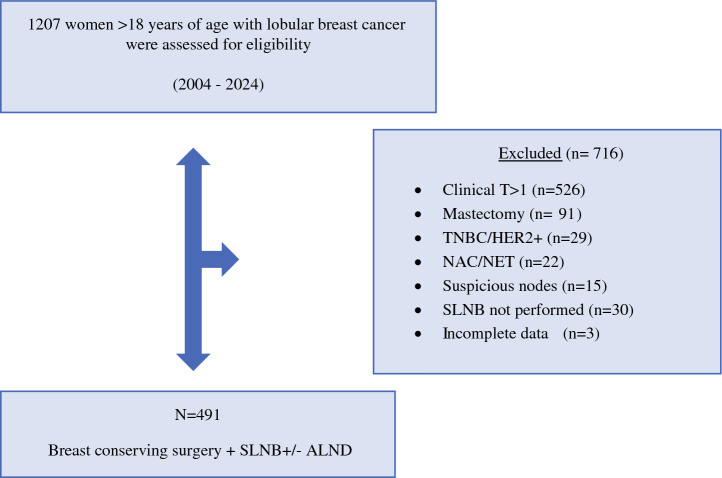
Study flow diagram. TNBC, triple-negative breast cancer; SLNB, sentinel lymph node biopsy; ALND, axillary lymph node dissection; NAC, neoadjuvant chemotherapy; NET, neoadjuvant endocrine therapy

This retrospective observational study was conducted at Fondazione Policlinico Universitario Agostino Gemelli IRCCS in Rome, Italy. Medical records of 1207 patients with histologically confirmed ILC who underwent surgery between 2004 and 2024 were reviewed. The study included 491 patients with estrogen receptor (ER)-positive/human epidermal growth factor receptor 2 (HER2)-negative tumors, clinical stage T1 disease, and clinically node-negative status (Fig. 1).

The study was approved by the Lazio-3 Ethics Committee on 25 September 2025 (ID: 7928), and all the patients provided informed consent for surgery and use of clinical data. The study was conducted in accordance with the Strengthening the Reporting of Observational Studies in Epidemiology (STROBE) guidelines for observational studies to ensure transparent reporting of study design, patient selection, data collection, and statistical analysis.

### Inclusion and Exclusion Criteria

The study population consisted of patients with histologically confirmed ILC who met well-defined inclusion criteria. Eligible cases were limited to tumors displaying a luminal phenotype, characterized by ER–positive and HER2-negative status. Only patients with a clinical tumor stage T1 and a clinically node-negative axilla were included. Nodal negativity was established through physical examination and axillary ultrasound, then corroborated by MRI findings. All patients were required to be 18 years of age or older and to have no evidence of distant metastases at the time of diagnosis. Moreover, inclusion required that patients had undergone breast-conserving surgery combined with SLNB as the initial surgical approach. Complete clinical, imaging, and histopathologic data, including histologic subtype classification (classic, solid, pleomorphic, alveolar, and mixed invasive lobular carcinoma) and definitive nodal staging, had to be available for all cases.

Patients were excluded from the study if they had received neoadjuvant chemotherapy or primary endocrine therapy. Individuals presenting with primary tumors larger than 2 cm (clinical stage T2 or higher) or with clinically or radiologically suspicious axillary lymph nodes requiring preoperative needle biopsy also were excluded. Given the strict selection of cT1 luminal ER-positive/HER2-negative patients undergoing upfront breast-conserving surgery without neoadjuvant therapy, only a limited number of cases (*n* = 15) presented with radiologically suspicious axillary nodes and were therefore excluded. Additional exclusion criteria ruled out a history of breast cancer, the presence of distant metastases at diagnosis, and cases in which mastectomy was performed instead of breast-conserving surgery. Finally, patients with incomplete clinical, imaging, or pathologic data were excluded from the analysis to ensure consistency and reliability of the results.

### Preoperative Assessment and Axillary Surgical Management

All the patients underwent standardized preoperative imaging, including axillary ultrasound and breast MRI with axillary evaluation, as part of the routine preoperative assessment.

All the patients underwent SLNB as part of the primary surgical management. Axillary lymph node dissection (ALND) was performed selectively based on nodal burden and contemporary clinical practice. For patients with limited nodal involvement (1–2 positive sentinel lymph nodes), omission of completion ALND was adopted in accordance with the progressive implementation of the ACOSOG Z0011 criteria.^15^

### Statistical Analysis

Descriptive statistics were used to summarize the distribution of clinicopathologic characteristics across nodal status groups. Categorical variables were compared using the chi-square test or Fisher’s exact test, as appropriate. Univariate analysis was first performed to assess associations between clinicopathologic factors and nodal status (node-negative vs node-positive [pN1mi–pN3]). Variables with a *p* value lower than 0.10 in the univariate analysis were subsequently entered into a multivariable logistic regression model to identify independent predictors of nodal metastases. The dependent variable was the presence of nodal metastases.

Odds ratios (ORs) and 95 % confidence intervals (CIs) were calculated. Model performance was assessed using the chi-square test for model fit and overall classification accuracy. A *p* value lower than 0.05 was considered statistically significant. All analyses were performed using IBM SPSS Statistics for Windows, version 27.0 (IBM Corp., Armonk, NY, USA).

## Results

### Patient and Tumor Characteristics

The analysis included 491 patients with early-stage, ER-positive/HER2-negative, clinically node-negative (cN0) ILC who underwent breast-conserving surgery and SLNB. The mean age at diagnosis was 60 years (range, 29–84 years), and the majority of the patients were postmenopausal (73.5 %). Progesterone receptor expression greater than 10 % was observed in 85.1 % of tumors, whereas 63.9 % of the cases demonstrated a Ki-67 proliferation index of 20 % or lower.

On final histopathologic examination, most tumors were confirmed as pT1 lesions, including pT1mic/pT1a in 18 patients (3.6 %), pT1b in 136 patients (27.7 %), and pT1c in 268 patients (54.7 %). A subset of tumors was upstaged to pT2, accounting for 69 patients (14.0 %). Importantly, no cases were upstaged to pT3. The mean pathologic tumor size was 14.5 mm (range, 1–50 mm).

Most of the tumors were histologic grade 2 (86.7 %), with grade 1 tumors accounting for 2.8 % and grade 3 tumors accounting for 10.3 % of the cases. Classic invasive lobular carcinoma was the predominant histologic subtype (67.8 %), followed by pleomorphic (16.3 %), alveolar (4.5 %), mixed (3.4 %), and other less common variants. These findings are detailed in Table 1.

**Table 1:** Clinicopathologic features by pathologic nodal status in 491 patients with invasive lobular carcinoma

	Total (*n* = 491) *n* (%)	pN0 (*n* = 392) *n* (%)	pN1mi (*n* = 19) *n* (%)		pN1 (*n* = 65) *n* (%)		pN2 (*n* = 8) *n* (%)	pN3 (*n* = 7) *n* (%)	*p* Value
Mean age: years (range)	60 (29–84)	60 (29–84)	61 (44–80)		57 (37–82)		59 (45–86)	57 (45–72)	
Menopausal status									
Premenopausal	130 (26.5)	102 (78.5)	5 (3.9)		18 (13.8)		3 (2.3)	2 (1.5)	0.96
Postmenopausal	361 (73.5)	290 (80.3)	14 (3.9)		47 (13.0)		5 (1.4)	5 (1.4)	
PR status									
<10	73 (14.9)	56 (76.7)	1 (1.4)		11 (15.0)		2 (2.8)	3 (4.1)	0.15
>10	418 (85.1)	336 (80.4)	18 (4.3)		54 (12.9)		6 (1.5)	4 (0.9)	
Ki67 status									
<20	314 (63.9)	252 (80.2)	12 (3.9)		38 (12.1)		7 (2.2)	5 (1.6)	0.56
>20	177 (36.1)	140 (79.1)	7 (4.0)		27 (15.3)		1 (0.5)	2 (1.1)	
Grading									
G1	14 (2.8)	12 (85.8)	1 (7.1)		1 (7.1)		0	0	0.14
G2	426 (86.7)	341 (80.0)	12 (2.8)		60 (14.1)		7 (1.7)	6 (1.4)	
G3	51 (10.3)	39 (76.5)	6 (11.8)		4 (7.7)		1 (2.0)	1 (2.0)	
Subtype									
Classic	333 (67.8)	276 (82.9)	11 (3.3)		40 (12.0)		5 (1.5)	1 (0.3)	0.15
Solid	11 (2.3)	10 (91.0)	1 (9.0)		0		0	0	
Pleomorphic	80 (16.3)	58 (72.5)	4 (5.0)		12 (15.0)		2 (2.5)	4 (5.0)	
Alveolar	22 (4.5)	16 (72.8)	0		4 (18.2)		1 (4.5)	1 (4.5)	
Mixed	17 (3.4)	11 (64.8)	2 (11.7)		4 (23.5)		0	0	
Others	28 (5.7)	21 (75.0)	1 (3.6)		5 (17.8)		0	1 (3.6)	
Mean T size: mm									
(range)	14.5 (1–50)	13.8 (1–40)	17.3 (7–50)		16.9 (5–50)		16.1 (8–20)	17.8 (9–30)	
Pathologic tumor size									
pT1mic/pT1a	18 (3.6)	16 (89.0)	1 (5.5)		1 (5.5)		0	0	**0.004**
pT1b	136 (27.7)	125 (92.0)	2 (1.5)		6 (4.3)		2 (1.5)	1 (0.7)	
pT1c	268 (54.7)	204 (76.2)	11 (4.1)		44 (16.4)		6 (2.2)	3 (1.1)	
pT2	69 (14.0)	47 (69.1)	5 (7.4)		14 (19.1)		0	3 (4.4)	
ALND									
No	421 (85.7)	372 (88.4)	16 (3.8)		33 (7.8)		0	0	
Yes	70 (14.3)	20 (28.6)	3 (4.3)		32 (45.6)		8 (11.5)	7 (10.0)	

PR, progesterone receptor; ALND, axillary lymph node dissection

Value shown in bold indicate statistical significance (*p* 0.05).

### Axillary Nodal Status

Pathologic nodal status was as follows: 392 patients (79.8 %) were confirmed as node-negative (pN0), 19 (3.9 %) had micrometastases (pN1mi), 65 (13.3 %) had 1 to 3 positive nodes (pN1), 8 (1.6 %) had 4 to 9 positive nodes (pN2), and 7 (1.4 %) had 10 or more positive nodes (pN3). Overall, 99 patients (20.2 %) harbored axillary nodal metastases (pN1mi–pN3).

Overall, ALND was performed for 70 patients (14.3 %). Among the 421 patients who did not undergo ALND, 372 (88.4 %) were classified as pN0, 16 (3.8 %) as pN1mi, and 33 (7.8 %) as pN1, with no cases of pN2 or pN3 disease. Among the patients who underwent ALND (*n* = 70), 20 (28.6 %) were pN0, 3 (4.3 %) were pN1mi, 32 (45.7 %) were pN1, 8 (11.4 %) were pN2, and 7 (10.0 %) were pN3. Notably, ALND for patients ultimately classified as pN0 was mainly attributable to intraoperative failure of sentinel lymph node mapping or historical practice patterns during the earlier years of the study period, before the widespread adoption of Z0011-based axillary de-escalation.

Among the 111 patients age ≥ 70 years, 18 (18.0 %) were found to have axillary nodal metastases. For 33 (7.8 %) patients with one or two positive lymph nodes treated between 2017 and 2024, ALND was not performed. No cases of ALND omission were observed before 2017. Axillary management decisions were made within a multidisciplinary framework and reflected evolving guideline-based practice during the study period.

### Factors Associated With Nodal Positivity

Pathologic tumor size remained significantly associated with axillary nodal involvement (*p* = 0.004). Increasing nodal positivity rates were observed across increasing pathologic tumor size categories. Tumors classified as pT1c demonstrated higher rates of nodal involvement than smaller pT1mic/pT1a and pT1b lesions. These findings indicate a progressive relationship between tumor size and axillary tumor burden despite exclusion of clinically larger tumors.

Histologic subtype demonstrated variability in nodal involvement patterns. Pleomorphic invasive lobular carcinoma showed higher rates of nodal positivity than the classic subtype, although this association did not reach statistical significance in univariable analysis (*p* = 0.15). Similarly, histologic grade was not significantly associated with nodal status (*p* = 0.14), with grade 3 tumors not demonstrating a disproportionate increase in nodal involvement compared with grade 1 or 2 tumors.

Proliferative activity, as assessed by Ki-67, was not significantly associated with nodal positivity in the univariable analysis (*p* = 0.56). Progesterone receptor expression also did not demonstrate a significant association with axillary nodal involvement (*p* = 0.15).

Neither age at diagnosis nor menopausal status was associated with nodal involvement in our analysis. Similarly, progesterone receptor status and histologic grade did not demonstrate independent associations with axillary nodal positivity.

### Multivariable Analysis

In multivariable logistic regression analysis (Table 2), with adjustment for age, menopausal status, tumor size, histologic grade, histologic subtype, and Ki-67, pathologic tumor size remained independently associated with axillary nodal positivity. Tumors upstaged to pT2 on final pathology demonstrated a higher likelihood of nodal involvement than tumors ≤pT1c (OR, 2.03; 95 % CI, 1.11–3.70; *p* = 0.021).

**Table 2:** Multivariable logistic regression analysis of predictors of axillary nodal metastases (pN1mi–pN3) in 491 patients

	OR	95 % CI	*p* Value
Age (≥50 years)	0.68	0.18–2.57	0.565
Postmenopausal status	1.10	0.30–3.98	0.887
Tumor size (pT2 vs ≤pT1c)	**2.03**	**1.11–3.70**	**0.021**
Histologic grade (G3 vs G1–2)	1.32	0.59–2.96	0.495
Histologic subtype (pleomorphic vs classic)	1.82	0.99–3.33	0.054
Ki-67 (>20 %)	**0.51**	**0.29–0.88**	**0.015**

OR, odds ratio; CI, confidence interval

Values shown in bold indicate statistical significance (*p* 0.05).

A level of Ki-67 greater than 20 % was independently associated with a lower probability of nodal involvement (OR, 0.51; 95 % CI, 0.29–0.88; *p* = 0.015). Histologic subtype (pleomorphic vs classic) showed a borderline association with nodal positivity (OR, 1.82; 95 % CI, 0.99–3.33; *p* = 0.054). Age, menopausal status, and histologic grade were not independently associated with axillary nodal involvement.

## Discussion

In our series, 99 (20.2 %) of 491 patients with early-stage, ER-positive/HER2-negative ILC and clinically node-negative axilla were found to have pathologic nodal metastases (pN1mi–pN3) at definitive surgery. Despite careful preoperative assessment with both ultrasound and MRI, approximately one in five patients presented with occult nodal metastases, underscoring the limited sensitivity of current imaging methods in accurately identifying nodal involvement in ILC.

The intrinsic challenges of axillary imaging in ILC complicate the safe omission of SLNB. Schumacher et al.^16^ reported that in 349 stages I to III ILC patients, 38 % of those with advanced nodal disease (pN2–pN3) were misclassified as cN0 on imaging despite MRI showing the highest sensitivity (65 %) followed by ultrasound (42 %) and mammography (10 %). These findings reinforce that imaging alone is insufficient for axillary staging in ILC and that SLNB remains essential, particularly for guiding adjuvant therapy.

Emerging methods, such as [18F]FDG PET/MRI, have shown promise in detecting nodal metastases in early breast cancer.^17^ However, lobular carcinomas represented a minority in these studies, and the infiltrative often hypometabolic nature of ILC may limit the accuracy of fluorodeoxyglucose (FDG)-based imaging.

The unique behavior of ILC in the axilla is further supported by Adachi et al.^18^ who demonstrated a higher frequency of non-sentinel lymph node metastases in ILC than in IDC when the sentinel node was positive. This suggests that nodal disease in ILC is often diffuse and unpredictable, indicating the need for precise surgical assessment even in cN0 patients. Consistently, Mukhtar et al.^19^ reported that accurate preoperative axillary staging in ILC remains difficult due to the tumor’s subtle and diffuse infiltration pattern, which may escape both clinical and imaging detection.

In older patients, the omission of SLNB in selected ILC cases may be safe. Carleton et al.^20^ found no significant difference in locoregional recurrence-free survival among women ≥70 years old with early-stage, ER-positive/HER2-negative, cN0 breast cancer, including those with ILC, whether SLNB was performed or omitted.

In our cohort, menopausal status was not associated with axillary nodal involvement (*p* = 0.96). In addition, a subgroup analysis of patients age ≥70 years showed that 18 % harbored nodal metastases, a proportion that did not differ meaningfully from the overall rate observed in the study population.

Collectively, these findings indicate that neither menopausal status nor chronological age alone reliably predicts nodal involvement in invasive lobular carcinoma and therefore should not be used in isolation to guide axillary surgical decision-making Among the variables analyzed, tumor size emerged as the strongest predictor of nodal burden. Larger tumors were significantly more common in node-positive groups, particularly among those with four or more positive lymph nodes. This finding, although somewhat expected, reinforces the well-established notion that greater tumor size is directly associated with increased axillary spread, even in luminal A-like tumors.^21^

As expected, after the cohort was restricted to patients with clinical T1 disease, a measurable rate of pathologic upstaging was observed. On final histopathologic examination, 69 patients (14.0 %) were upstaged to pT2, underscoring the intrinsic limitations of preoperative tumor size assessment in invasive lobular carcinoma. This finding was not unexpected because ILC is characterized by a diffuse growth pattern and ill-defined tumor margins, which may lead to underestimation of true tumor extent on imaging and clinical evaluation.^22^

Histologic subtype did not demonstrate a statistically significant association with axillary nodal status in the analysis. However, pleomorphic and mixed variants showed a numerically higher prevalence of nodal involvement than classic invasive lobular carcinoma. Although classic ILC was more frequently observed among node-negative patients, these more aggressive variants tended to be overrepresented in higher nodal categories. Although this trend did not reach statistical significance, it suggests potential biologic heterogeneity within the ILC spectrum that may warrant consideration when axillary management strategies are evaluated.^23^

Recent trials have promoted selective de-escalation of axillary surgery in early-stage breast cancer. However, the 2025 St. Gallen International Consensus highlighted that these trials included only a small proportion of ILC patients, and a majority of panelists (60 vs 40 %) recommended against omitting SLNB in this histologic subtype.^24^ National guidelines differ: the 2025 AGO Breast Commission integrates SOUND and INSEMA data, but does not distinguish between ductal and lobular cancers, recommending SLNB omission based on low-risk criteria regardless of histology.^25^

In the SOUND and INSEMA trials, which enrolled predominantly IDC, sentinel lymph node positivity rates were 13.7 % and 15 %, respectively, whereas in our pure ILC population, the rate reached 20.2 % (Table 3). However, the current study aimed to underscore that ILC constitutes a biologically distinct entity, characterized by unique pathophysiologic features that limit the applicability of axillary de-escalation strategies traditionally developed for IDC. Even in small, luminal-type tumors, lobular histology appears to retain a non-negligible risk of axillary metastasis, which may not be adequately predicted by conventional staging tools.^26^

**Table 3:** Lymph node positivity rates of SLNB omission trials and the current study

	Histologic subtype	No. of patients	SLNB positivity rate (%)
SOUND trial^13^	Predominantly IDC (77.8 %)	708	13.7
INSEMA trial^14^	Mostly NST (72.6 %)	3854	15.0
Current study (Gemelli ILC cohort, 2025)	Pure ILC	491	20.2

SLNB, sentinel lymph node biopsy; IDC, invasive ductal carcinoma, NST, non-special type, ILC, invasive lobular carcinoma

In addition, it should be considered that the INSEMA trial included a small proportion of patients with clinical T2 tumors, whereas in our analysis, similar to the SOUND trial, only tumors clinically assessed as ≤ 2 cm were included, based on preoperative ultrasound and MRI. Despite this strict selection of clinically small tumors, a pathologic upstaging rate of 14 % was observed, further highlighting the intrinsic challenges of accurately estimating tumor extent in invasive lobular carcinoma.

Importantly, 3 % of the patients harbored four or more positive nodes, a nodal burden that would significantly influence adjuvant treatment decisions, including the indication for chemotherapy or regional nodal irradiation. These data suggest that omitting SLNB in this subgroup may result in understaging and potential undertreatment.^27,28^

Limitations of this study included its retrospective, single-center design which may have introduced selection bias and may limit generalizability. The long inclusion period may have encompassed evolving imaging, surgical, and pathologic practices. Finally, only patients undergoing breast-conserving surgery were included, potentially underrepresenting the full spectrum of early-stage ILC.

## Conclusion

This study, based on a large and homogeneous cohort of patients with early-stage, ER-positive/HER2-negative ILC, demonstrates that nodal metastases remain frequent despite clinically negative axilla and optimal preoperative imaging. Nearly one in five patients harbored occult nodal disease, emphasizing the limited sensitivity of current imaging methods in ILC and the continued diagnostic value of SLNB. The data highlight that ILC behaves as a biologically distinct entity with a significant and often unpredictable risk of nodal involvement even in small, low-grade, luminal tumors.

Although ongoing de-escalation trials support SLNB omission in selected low-risk breast cancers, their applicability to ILC remains uncertain due to its underrepresentation and distinct biologic behavior. Future prospective, multicenter studies specifically designed for ILC are warranted to refine axillary management strategies and safely identify patients who may benefit from surgical de-escalation without compromising oncologic outcomes.
